# TMAF-YOLO: A Lightweight Model for In Situ Detection of Tomato Maturity and Defective Fruits in Greenhouses

**DOI:** 10.3390/s26154950

**Published:** 2026-08-05

**Authors:** Chenxiao Huang, Linran He, Wentao Huang, Xiaoshuan Zhang

**Affiliations:** College of Engineering, China Agricultural University, Beijing 100083, China; 2024307150110@cau.edu.cn (C.H.);

**Keywords:** tomato maturity detection, YOLOv8n, lightweight model, class imbalance, greenhouse vision detection

## Abstract

Accurate tomato maturity detection is essential for harvesting decisions, quality grading, and postharvest handling in greenhouse production. However, leaf occlusion, fruit overlap, complex backgrounds, illumination variation, and subtle color differences between adjacent maturity stages limit real-time detection performance. To improve accuracy and deployment efficiency, this study proposes TMAF-YOLO, a lightweight detection model based on YOLOv8n. In this model, LGhostConv replaces selected convolutional structures to reduce redundant computation. A tomato maturity-aware aggregation fusion module, termed TMAF, is introduced to enhance color, texture, and local structural feature representation in the detection branches. MA-CB Focal Loss is used to improve learning under class imbalance and hard-sample conditions. Experimental results showed that TMAF-YOLO achieved Precision, Recall, mAP50, and mAP50-95 values of 0.889, 0.873, 0.954, and 0.776, respectively. The model contained 2.647 M parameters and required 7.3 GFLOPs, with an inference speed of 209.030 FPS. Compared with YOLOv8n and heavier detection models, TMAF-YOLO achieved higher detection accuracy with fewer parameters and lower computational cost. It also outperformed YOLOv11n and YOLOv12n in detection accuracy while maintaining real-time inference performance. These results indicate that the proposed model is suitable for real-time greenhouse tomato maturity detection and can support automated harvesting and grading.

## 1. Introduction

Tomato is an important vegetable crop in protected agriculture. Its maturity directly affects harvesting time, transport management, postharvest ripening, quality grading, and commercial value. In greenhouse environments, tomato fruits are often affected by leaf occlusion, fruit overlap, uneven illumination, and scale variation. Manual maturity assessment is inefficient and easily influenced by subjective experience. Therefore, a rapid, stable, and non-contact detection method is needed to support automated harvesting, grading management, and robotic visual perception in protected agriculture [1,2,3].

Among deep learning detection methods, region-based convolutional neural network (R-CNN) models and their variants were among the earlier methods applied to fruit and vegetable detection and counting tasks. An improved Faster R-CNN model, MatDet, was proposed for multi-object tomato maturity detection in complex scenarios [1]. The model used a 50-layer residual network (ResNet-50), region-of-interest alignment (RoIAlign), and a path aggregation network (PANet) to improve detection under occlusion, overlap, and illumination variation, achieving a mean average precision (mAP) of 96.14%. Faster R-CNN has also been used for tomato-related detection tasks. Highly occluded immature tomatoes on plants were detected using Faster R-CNN, with an average precision of 87.83% [4]. Greenhouse tomato flowers were detected and counted using Faster R-CNN, achieving an average precision of 96.02% [5]. These methods have strong localization ability and can provide stable target region information. However, their model structures are relatively complex, and their inference speed and lightweight deployment ability remain limited.

With the development of Transformer architectures, end-to-end detectors such as Detection Transformer (DETR), DETR with Improved deNoising anchOr boxes (DINO), and Real-Time Detection Transformer (RT-DETR) have been introduced into object detection tasks. DETR formulates object detection as a set prediction problem [6]. DINO improves the convergence and detection performance of DETR-like models through denoising training and query optimization [7]. RT-DETR further improves the Transformer detection structure for real-time detection requirements [8]. In fruit and vegetable maturity recognition, PDSI-RTDETR was proposed for tomato maturity detection. Compared with the original RT-DETR, its mAP50 and mAP50-95 increased by 3.9 and 4.1 percentage points, respectively [9]. An improved RT-DETR was also applied to fruit ripeness detection, indicating the potential of Transformer detectors in global feature modeling [10]. However, these models usually have higher training costs, larger parameter sizes, and greater hardware requirements.

In contrast, You Only Look Once (YOLO) series models provide a better balance between detection accuracy and inference speed. They have become common methods for fruit and vegetable detection and maturity recognition. A lightweight improved YOLOv5 model was proposed for real-time tomato detection and mobile deployment [2]. Compared with the original YOLOv5s, the number of parameters and floating-point operations (FLOPs) were reduced by 78% and 84.15%, respectively, while maintaining high detection accuracy. RGB-D information was combined with YOLOv5 to detect and count greenhouse tomato clusters [3]. MTD-YOLO was proposed for multitask detection of cherry tomato fruits, clusters, and maturity [11]. The model achieved an overall detection score of 86.6% and an average inference time of 4.9 ms. An improved YOLOX model was proposed for tomato ripeness and stem recognition, achieving an mAP of 92.17% and improving recognition under sample imbalance and scale variation [12]. YOLOv8 was combined with a lightweight Swin Transformer for strawberry ripeness detection [13]. Compared with YOLOv5s, CenterNet, and the Single Shot MultiBox Detector (SSD), the mAP50 increased by 1.6%, 33.5%, and 3.4%, respectively. These results indicate that improved YOLO models are suitable for fine-grained fruit maturity recognition. In addition, recent YOLO-based studies on tomato detection and ripening recognition have improved detection performance using attention mechanisms, lightweight structures, multiscale feature fusion, and postprocessing optimization [14,15,16,17].

Existing studies have verified the effectiveness of deep learning methods in fruit and vegetable detection and maturity recognition. However, further improvement is still needed for in situ greenhouse tomato detection. First, the color transition between semi-mature and mature tomatoes is continuous, making fine-grained classification difficult. Second, leaf occlusion, fruit overlap, and illumination variation weaken maturity-related feature representation. Third, defective samples are relatively limited, and conventional classification losses may underemphasize minority and hard samples. Previous studies have shown that Focal Loss and Class-Balanced Loss can adjust the loss weights of hard and imbalanced samples, thereby improving the learning ability for long-tailed classes and difficult samples [18,19]. To address these issues, this study proposes TMAF-YOLO based on YOLOv8n.

The main contributions of this study are as follows.

(1) A lightweight tomato maturity detection model was constructed for complex greenhouse scenarios. Inspired by the low-cost feature generation strategy in GhostNet, an LGhostConv module was designed and embedded in key positions of the Backbone and Neck [20]. This design reduces the number of parameters and computational cost while maintaining detection performance.
(2) A tomato maturity-aware aggregation fusion module, termed TMAF, was proposed. This module was designed to address continuous color transitions between adjacent maturity stages, weak local texture differences, and insufficient feature representation caused by occlusion and overlap. By integrating local texture enhancement and channel-spatial recalibration, TMAF strengthens maturity-related feature representation.
(3) MA-CB Focal Loss was developed to improve learning under class imbalance and hard-sample conditions. By combining class-balanced weights with the Focal Loss modulation term, the loss enhances learning for minority and difficult samples, including defective fruits and adjacent maturity stages.

## 2. Materials and Methods

### 2.1. Overall Method Design

**Figure 1:**
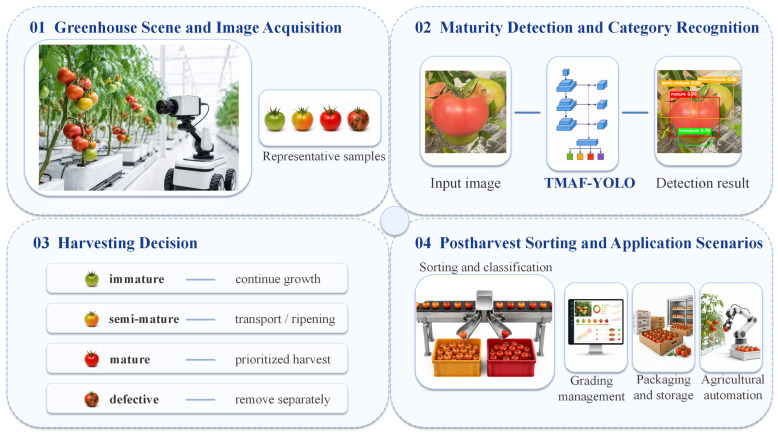
Overall workflow of greenhouse tomato maturity detection.

This study focuses on in situ tomato maturity detection before greenhouse harvesting. An overall application workflow was constructed, including greenhouse image acquisition, fruit detection and category recognition, harvesting decision-making, and postharvest grading management. As shown in Figure 1, tomato images were first acquired under natural growth conditions in the greenhouse. Then, the developed detection model was used to identify fruit locations, maturity stages, and defective fruits. Finally, the detection results were used to support harvesting time determination, defective fruit screening, and postharvest grading and quality management. It should be noted that this study mainly focuses on greenhouse tomato maturity and defective fruit detection. The harvesting, grading, and management processes in the figure are used to illustrate the potential application scenarios of the proposed model.

### 2.2. Dataset Construction and Annotation

The self-collected images used in this study were acquired in a tomato greenhouse at the Tianjin Academy of Agricultural Sciences, China, from 10 to 17 January 2026. Image acquisition was conducted between 9:00 a.m. and 12:00 p.m. using a HUAWEI Mate 60 smartphone (Huawei Device Co., Ltd., Dongguan, China) in automatic shooting mode. Exposure, focus, ISO sensitivity, and shutter speed were adjusted automatically by the built-in camera system. The shooting distance ranged from approximately 0.5 to 2.0 m, and the image resolution was 1280 × 760 pixels. Images were captured under naturally varying greenhouse illumination; illuminance was not measured instrumentally.

**Figure 2:**
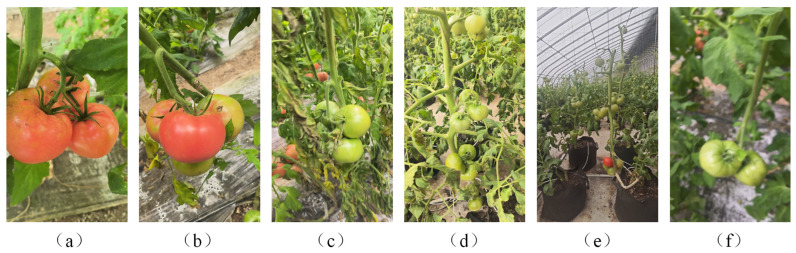
Representative tomato images acquired under typical greenhouse conditions: (**a**) mature fruits; (**b**) adjacent fruits at different maturity stages; (**c**) fruit overlap and foliage interference; (**d**) leaf and branch occlusion; (**e**) small targets in a relatively long-range view; and (**f**) local image blur.

To represent practical greenhouse conditions, images were acquired from different viewing angles and distances. The collected scenes included fruits at different maturity stages and scales, together with leaf and branch occlusion, fruit overlap, dense fruit distribution, small targets, complex backgrounds, and local image blur. Representative examples of self-collected images are shown in Figure 2.

A total of 596 valid images were collected from the greenhouse. Because defective fruits were relatively scarce, 33 additional defective-fruit images were selected from the Tomato Fruit Diseases Dataset available on Kaggle (https://www.kaggle.com/datasets/profnourasemary/tomato-dataset, accessed on 1 July 2026). The additional images mainly consisted of tomato fruits exhibiting visible disease symptoms, together with a smaller number showing physical damage, decay, cracking, or other abnormal surface characteristics. The selected public images were annotated according to the same criteria as the self-collected images.

The 596 self-collected images were divided at the original-image level into training, validation, and test subsets containing 470, 63, and 63 images, respectively. The 33 public images were added exclusively to the training set to increase the number and diversity of defective-fruit samples. Consequently, the final training, validation, and test sets contained 503, 63, and 63 images, respectively. The validation and test sets therefore consisted entirely of self-collected greenhouse images.

The annotated tomato instances were divided into four categories: immature, semi-mature, mature, and defective. The first three categories represent successive normal maturity stages based primarily on fruit color and visible appearance. The defective category includes fruits with visible disease spots, physical damage, decay, cracking, or other abnormal surface characteristics and was treated as an independent appearance category rather than as a normal maturity stage.

All tomato instances were manually annotated with bounding boxes using LabelMe v6.0.0. The original annotations were stored in JSON format and subsequently converted into the format required by the YOLO detection framework. Partially occluded or locally blurred fruits were retained when their categories and principal visible boundaries could be reliably determined. Instances were excluded only when severe occlusion, insufficient visible information, or ambiguous appearance prevented consistent annotation. All annotations were reviewed according to uniform class definitions to ensure labeling consistency.

The final dataset contained 629 images and 2505 annotated tomato instances, including 1170 immature, 788 semi-mature, 304 mature, and 243 defective instances. Data augmentation was performed after dataset partitioning and was applied only to the training set. The validation and test sets were not augmented. The training set was used for model parameter optimization, the validation set for training monitoring and model selection, and the test set exclusively for final performance evaluation. The image and instance distributions before data augmentation are summarized in Table 1.

### 2.3. Construction of the TMAF-YOLO Model

#### 2.3.1. TMAF-YOLO: An Improved YOLOv8n Model

**Figure 3:**
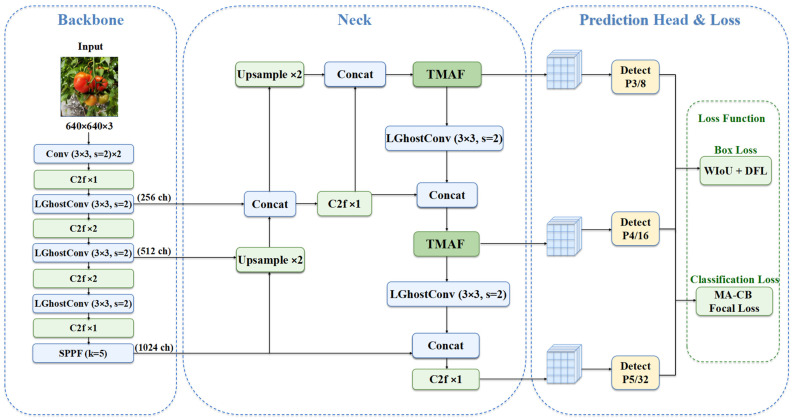
Overall architecture of TMAF-YOLO.

The proposed TMAF-YOLO uses YOLOv8n as the baseline model. It is improved from three aspects: lightweight feature extraction, maturity-related feature fusion, and classification loss optimization. The overall architecture is shown in Figure 3. After the original image is input into the network, multiscale features are first extracted by the Backbone, in which selected convolutional structures are replaced by LGhostConv. Then, features at different scales are fused in the Neck, and TMAF modules are added to the P3/8 and P4/16 branches. Finally, the model outputs prediction results through three detection heads at P3/8, P4/16, and P5/32 scales. During training, the model is optimized using a joint loss function composed of WIoU, DFL, and MA-CB Focal Loss. The specific designs of LGhostConv, TMAF, and MA-CB Focal Loss are introduced in the following sections.

#### 2.3.2. Lightweight Feature Extraction Module: LGhostConv

To reduce redundant computation during feature extraction and multiscale feature fusion, a lightweight Ghost convolution module, termed LGhostConv, was introduced into selected downsampling layers of the backbone and neck. Similar to GhostConv, LGhostConv generates output features through primary and complementary feature branches. However, it replaces the dense spatial convolution in the primary branch with a depthwise-separable operation and employs directional depthwise convolutions in the complementary branch.

Given an input feature map X with $C_{in}$ channels, LGhostConv first applies a k×k depthwise convolution with stride s for spatial feature extraction and downsampling, followed by a 1×1 pointwise convolution for channel interaction. This process generates approximately half of the required output channels as the primary features. The remaining channels are generated by dividing the primary features into two groups and applying 1×3 and 3×1 depthwise convolutions to capture horizontal and vertical information, respectively. The directional features are then fused with the primary features through a parameter-free residual connection and concatenated to form the final output.

Ignoring bias terms, batch-normalization parameters, and activation operations, the number of parameters in LGhostConv is expressed as follows:

(1) $$P_{LGhostConv}=k^{2}C_{in}+C_{in}m+3n$$

where m = ⌈$C_{out}$/2⌉ and n = $C_{out}$ − m denote the numbers of primary and complementary feature channels, respectively. In comparison, a standard k×k convolution requires $k^{2}C_{in}C_{out}$ parameters. Therefore, LGhostConv reduces model complexity by replacing dense spatial channel interactions with depthwise spatial filtering, pointwise channel projection, and low-cost directional feature generation. Its effects on detection accuracy, model size, computational complexity, and inference speed are evaluated in Section 3.3.

#### 2.3.3. Tomato Maturity-Aware Aggregation Fusion Module: TMAF

**Figure 4:**
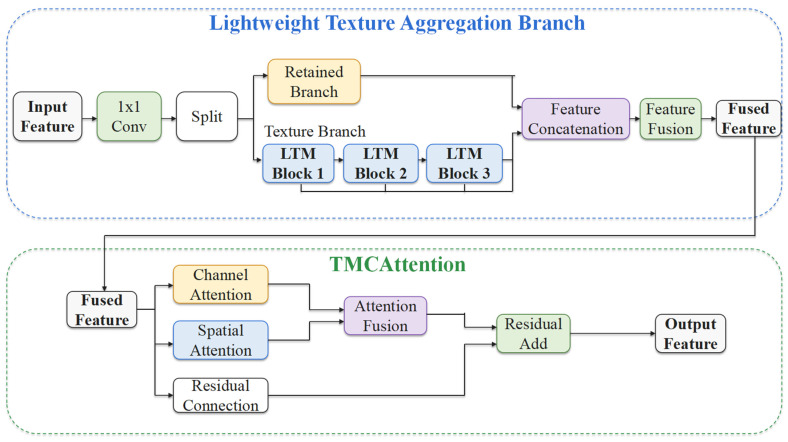
Structure of the TMAF module.

To strengthen the representation of maturity- and defect-related cues in the fused neck features, a tomato maturity-aware aggregation fusion module, termed TMAF, was designed. TMAF is embedded in the P3 and P4 feature fusion paths of the YOLOv8n neck, where relatively high-resolution features are retained for fine-grained maturity and defect detection. The original P5 path is preserved to limit the additional computational cost. As illustrated in Figure 4, TMAF comprises a lightweight texture aggregation branch and a TMCAttention submodule.

Let the input feature be X, with a size of $X\in R^{B\times C\times H\times W}$, where B, C, H, and W denote the batch size, number of channels, feature map height, and feature map width, respectively. The input feature first passes through a 1×1 convolution for channel mapping. It is then split along the channel dimension into a retained branch and a texture branch:

(2) $$X_{1},X_{2}=Split(Conv_{1\times 1}(X))$$

where $X_{1}$ is the retained branch, which maintains the direct transmission of input information. $X_{2}$ is the texture branch, which passes through three LTMBlocks in sequence to obtain texture-enhanced features at different levels, denoted as $T_{1}$, $T_{2}$, and $T_{3}$. Each LTMBlock consists of a 1×1 convolution, a 3×3 depthwise convolution, a 5×5 depthwise convolution, and a 1×1 convolution. The depthwise convolutions are used to supplement local texture and edge details. The 1×1 convolutions are used for channel adjustment and feature fusion.

Then, the retained branch $X_{1}$, and the texture branch outputs $T_{1}$, $T_{2}$, and $T_{3}$ are concatenated along the channel dimension. A 1×1 convolution is then used to complete feature fusion and obtain the fused feature F:

(3) $$=Conv_{1\times 1}(Concat(X_{1},T_{1},T_{2},T_{3}))$$

To further adjust maturity-related feature responses, TMCAttention is applied after feature fusion. This structure contains three branches: channel attention, spatial attention, and residual connection. Channel attention is used to adjust the importance of different feature channels. It helps the model focus on color, texture, and defect responses related to maturity discrimination. Spatial attention is used to highlight fruit region responses and reduce interference from irrelevant background features. The outputs of the two attention branches are denoted as $F_{c}$ and $F_{s}$, respectively.

Finally, the outputs of the channel attention branch and the spatial attention branch are fused and added to the residual branch. The output feature of the TMAF module is obtained as follows:

(4) $$Y=F+Conv_{1\times 1}(F_{c}+F_{s})$$

where Y denotes the output feature of the TMAF module. Through this design, TMAF introduces local texture enhancement, multibranch feature aggregation, and channel-spatial attention recalibration while preserving the original fused features. This improves the feature representation ability of the P3 and P4 branches for key categories, including semi-mature, mature, and defective fruits.

#### 2.3.4. Loss Function Optimization: MA-CB Focal Loss

To alleviate class imbalance among tomato categories and reduce the dominance of easily classified samples, the original classification loss of YOLOv8n was replaced with maturity-aware class-balanced focal loss, termed MA-CB Focal Loss. This modification was applied only to the classification branch, whereas WIoU and DFL were retained for bounding-box regression. MA-CB Focal Loss combines effective-number-based class weighting with focal modulation to adjust the contributions of different categories and prediction elements during training.

Let $n_{c}$ denote the number of annotated instances belonging to class c in the training set. The class-balanced weight is defined as follows:

(5) $$\alpha_{c}=\frac{1-\beta }{1-\beta^{n_{c}}}$$

where β ∈ [0, 1) controls the rate at which the effective number of samples increases with class frequency. A value closer to 1 increases the sensitivity of the class-balanced weights to differences in class frequency. Preliminary experiments were conducted using β ∈ {0.99, 0.999, 0.9999}. Among these settings, β = 0.999 achieved the best overall detection performance and was therefore fixed throughout training.

The values of $n_{c}$ were calculated exclusively from the annotated instances in the training set before data augmentation; validation and test annotations were not used to construct the class weights.

For prediction element i and class c, let $z_{ic}$ and $y_{ic}$ denote the predicted logit and target score, respectively, and let $p_{ic}$ = σ($z_{ic}$). The probability assigned to the target state is calculated as $p_{t,ic}$ = $y_{ic}$_c_$p_{ic}$ + (1 − $y_{ic}$)(1 − $p_{ic}$).

The MA-CB Focal Loss is then expressed as follows:

(6) $$L_{MA\text{-}CB}=\frac{1}{S}\sum_{i=1}^{N}\sum_{c=1}^{C}\alpha_{c}(1-p_{t,ic}{)}^{\gamma }l_{BCE}(z_{ic},y_{ic})$$

where N and C denote the number of prediction elements and tomato categories, respectively, and S is the sum of the target scores used for loss normalization. The term $l_{BCE}(z_{ic},y_{ic})$ denotes the binary cross-entropy loss computed directly from the prediction logit $z_{ic}$ and the target score $y_{ic}$. The focusing parameter γ controls the attenuation of easily classified elements. As γ increases, elements with high $p_{t,ic}$ contribute less to the classification loss, thereby increasing the relative contribution of difficult elements. Preliminary experiments were conducted using γ∈{1.0, 1.5, 2.0}. Among these settings, γ = 1.5 achieved the best overall detection performance and was therefore fixed throughout training.

The overall loss function of TMAF-YOLO is defined as

(7) $$L_{total}=\lambda_{box}L_{WIoU}+\lambda_{cls}L_{MA\text{-}CB}+\lambda_{dfl}L_{DFL}$$

where $L_{WIoU}$, $L_{MA\text{-}CB}$ and $L_{DFL}$ denote the WIoU-based bounding-box regression loss, the modified classification loss, and the distribution focal loss, respectively. The coefficients $\lambda_{box}$, $\lambda_{cls}$, and $\lambda_{dfl}$ are their corresponding loss gains.

### 2.4. Experimental Settings and Evaluation Metrics

#### 2.4.1. Hardware and Software Environment

All experiments in this study were conducted under the same hardware and software environment. The experimental platform used the Windows 11 operating system. The processor was an Intel Core i9-14900HX (Intel Corporation, Santa Clara, CA, USA), and the graphics card was an NVIDIA GeForce RTX 4060 Laptop GPU (NVIDIA Corporation, Santa Clara, CA, USA) with approximately 8 GB of memory. Model training, validation, and inference testing were all performed on this GPU to ensure comparability of experimental results among different models.

The software environment was built with Python 3.10.20. The deep learning framework used PyTorch 2.10.0 and TorchVision 0.25.0, and GPU acceleration was based on CUDA 12.8. The training, validation, and inference of YOLO series models were mainly implemented using the Ultralytics 8.4.42 framework. Image processing and numerical computation were performed using OpenCV 4.13.0, NumPy 2.2.6, and Scikit-learn 1.7.2.

#### 2.4.2. Training Hyperparameters

During model training, YOLOv8n was used as the baseline model. YOLOv8n, improved module combination models, and the final TMAF-YOLO model were compared under the same training conditions. All models used the same dataset division. The training, validation, and test sets were divided at a ratio of 8:1:1. The input image size was uniformly set to 640 × 640 to meet the requirements of batch training for YOLO models.

The number of training epochs was set to 150, and the batch size was set to 8. AdamW was used as the optimizer, with an initial learning rate of 0.01 and a weight decay coefficient of 0.0005. To improve training stability and support further convergence in the later training stage, a staged learning rate and weight decay adjustment strategy was used. The learning rate was reduced to 0.5 times its previous value every 30 epochs. The weight decay was reduced to 0.5 times its previous value every 50 epochs.

For the YOLO series models and the proposed improved models, the same training parameters were used. DETR, DINO, and R-CNN series models were initialized with public pretrained weights. For the R-CNN series models, a small number of shallow backbone parameters were frozen. All models were trained on the same dataset and hardware environment. Their final performance was evaluated on the independent test set.

#### 2.4.3. Evaluation Metrics

The following metrics were used to evaluate network performance. The evaluation covered both detection accuracy and model efficiency. For detection accuracy, Precision and Recall were used to measure the ability to suppress false detections and control missed detections, respectively. mAP50 represents the mean average precision of all classes at an IoU threshold of 0.5. It was used to evaluate target recognition ability under common detection conditions. mAP50-95 represents the average detection precision when the IoU threshold ranges from 0.5 to 0.95 with a step size of 0.05. It reflects localization accuracy and detection stability under stricter conditions.

For model efficiency, Param represents the number of model parameters and is used to measure model size and storage cost. FLOPs represent the number of floating-point operations and reflect computational complexity. FPS represents the number of image frames processed by the model per second. Infer time/ms represents the average inference time for a single image. FPS and Infer time/ms were jointly used to evaluate inference efficiency and provide a reference for real-time tomato maturity detection in greenhouse environments.

## 3. Results and Discussion

### 3.1. Training Process and Final Detection Result Analysis

#### 3.1.1. Training Loss Curve Analysis

**Figure 5:**
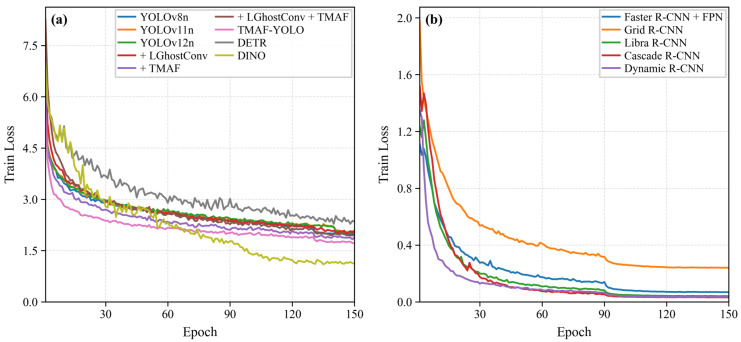
Training loss curves of different detection models. (**a**) One-stage and end-to-end detectors; (**b**) two-stage detectors.

To observe the training convergence of different models, the training loss curves were plotted, as shown in Figure 5. As the number of training epochs increased, the training losses of all models showed an overall decreasing trend. The curves gradually became stable in the later training stage. This indicates that all models could complete effective training on the dataset used in this study. It should be noted that different detection models have different network structures and loss function components. Therefore, the numerical scales of their training losses are not completely consistent. The curves are mainly used to analyze convergence trends and training stability, rather than to directly judge model performance.

#### 3.1.2. Validation mAP50 Curve Analysis

**Figure 6:**
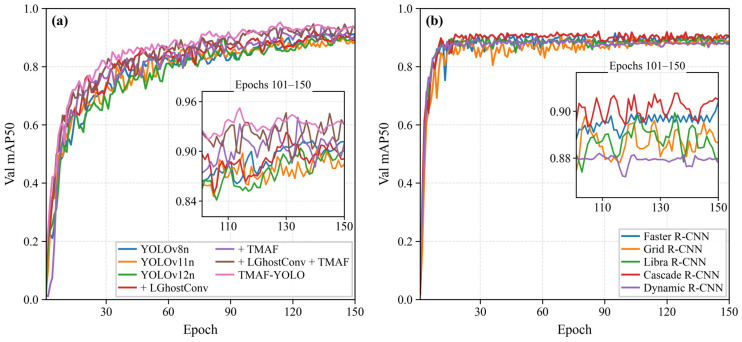
Validation mAP50 curves of different detection models. (**a**) One-stage and end-to-end detectors; (**b**) two-stage detectors.

To further analyze the performance changes in the models on the validation set, the validation mAP50 curves were plotted, as shown in Figure 6. The mAP50 values of all models increased rapidly in the early training stage and then gradually entered a stable stage. This indicates that the models gradually learned tomato localization and maturity classification features. In the later training stage, the curves remained generally stable, and no obvious performance degradation was observed. These results indicate that the training process in this study had good stability.

#### 3.1.3. Precision–Recall Curve Analysis

**Figure 7:**
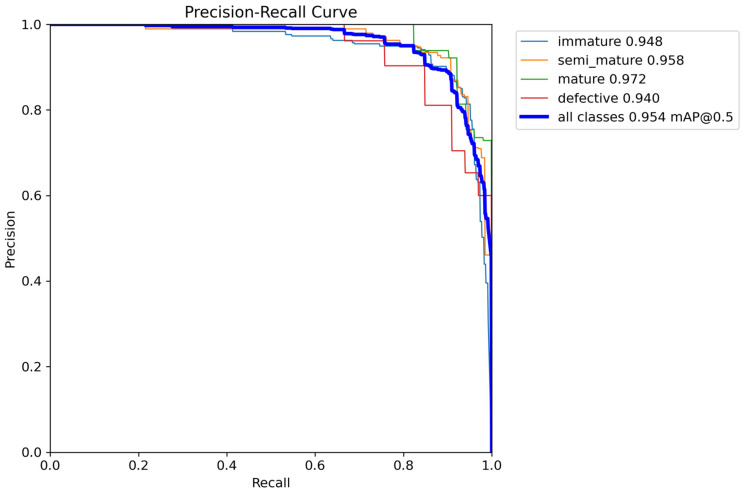
Precision–recall curve of TMAF-YOLO.

To analyze the relationship between precision and recall for different categories in the final model, the PR curve of TMAF-YOLO was plotted. The precision–recall curve is shown in Figure 7. The curves of the four categories were mainly located in the upper region of the PR space. This distribution shows that the model maintained good precision under high recall conditions. The AP values of immature, semi-mature, mature, and defective were 0.948, 0.958, 0.972, and 0.940, respectively. The overall mAP50 was 0.954, which corresponds to the mAP50 value reported in the evaluation results.

Among the four categories, mature had the highest AP value. This may be because mature tomatoes have a more distinct color and appearance features. The AP value of defective was slightly lower, possibly because of its smaller sample size and larger appearance variation. Overall, the PR curve results were consistent with the previous quantitative evaluation results. This further confirms that TMAF-YOLO achieved good detection performance across different categories.

#### 3.1.4. Confusion Matrix Analysis

To further observe misclassification among different categories, the normalized confusion matrices of YOLOv8n and TMAF-YOLO were compared, as shown in Table 2. For YOLOv8n, the correct recognition ratios for immature, semi-mature, mature, and defective were 0.87, 0.86, 0.79, and 0.89, respectively. For TMAF-YOLO, these values increased to 0.91, 0.91, 0.86, and 0.90, respectively.

The diagonal values of TMAF-YOLO improved for all four categories. The improvements for mature and semi-mature were more evident, indicating that the improved model enhanced the discrimination ability for adjacent maturity stages. The off-diagonal values show that a small amount of confusion still existed between semi-mature and mature. This is mainly related to the continuous tomato ripening process and unclear color and texture boundaries. The defective category also had a small number of misclassifications, which may be associated with large appearance variations and limited sample size.

### 3.2. Model Performance and Visualization Analysis

To evaluate the effectiveness of the proposed TMAF-YOLO model, it was compared with mainstream object detection methods. Under the same dataset division and experimental environment, YOLOv8n, YOLOv11n, YOLOv12n, DETR, DINO, Faster R-CNN, Grid R-CNN, Libra R-CNN, Cascade R-CNN, and Dynamic R-CNN were selected for comparison. These models cover one-stage detectors, end-to-end detectors, and two-stage detectors. This selection enables a broad comparison of different detection paradigms for greenhouse tomato maturity detection. In this study, the best model weights during training were selected using the validation set. The final detection performance of each model was then evaluated on the independent test set. The comparison results are shown in Table 3.

Table 3 shows that TMAF-YOLO achieved strong overall performance. Compared with the baseline YOLOv8n, the Precision of TMAF-YOLO increased from 0.858 to 0.889. Recall increased from 0.836 to 0.873, mAP50 increased from 0.916 to 0.954, and mAP50-95 increased from 0.744 to 0.776. These improvements demonstrate the effectiveness of the proposed modifications for tomato maturity detection. Meanwhile, TMAF-YOLO had 2.647 M parameters and 7.3 GFLOPs. Its average inference time was 4.784 ms, and its FPS reached 209.030. Thus, the model improved detection accuracy while maintaining a lightweight structure and real-time inference performance.

Although YOLOv11n and YOLOv12n are more recent detectors, they did not consistently outperform YOLOv8n on the present dataset. YOLOv11n achieved slightly higher Recall and mAP50-95 than YOLOv8n but lower Precision and mAP50, whereas YOLOv12n showed lower overall detection accuracy under the current settings. This result does not indicate that the newer architectures are intrinsically inferior. Their advantages on general-purpose benchmarks may not be fully realized on this relatively small and task-specific dataset, which contains substantial scale variation, fruit occlusion and overlap, local blur, and subtle visual differences among the four tomato categories. In contrast, TMAF-YOLO was specifically designed to strengthen high-resolution color, texture, and local structural features related to tomato maturity and defects. In addition, the same training configuration was used for all YOLO models to ensure a controlled comparison, although this configuration may not be optimal for each architecture.

DETR and DINO have strong global modeling capabilities, but their parameter counts reached 41.556 M and 47.393 M, respectively, and their average inference times were 41.777 ms and 33.152 ms, respectively. Among the two-stage detectors, Dynamic R-CNN and Grid R-CNN achieved Recall values of 0.885 and 0.873, respectively, indicating good target recall ability. However, the higher model complexity and inference latency of these models make them less favorable for lightweight real-time greenhouse deployment.

**Figure 8:**
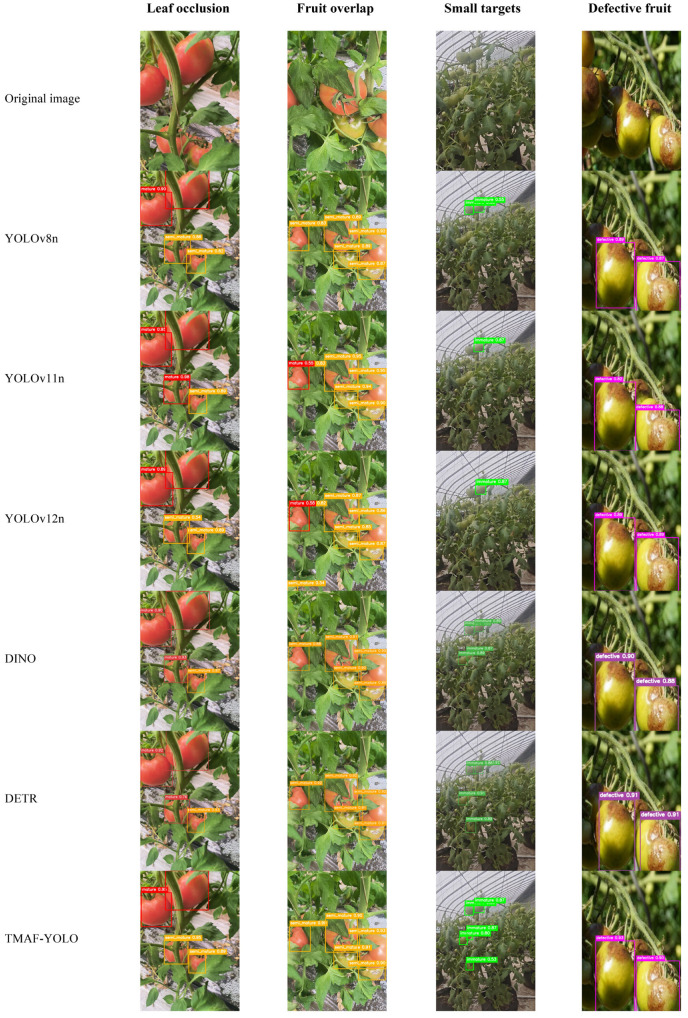
Detection results of one-stage and end-to-end detectors. Green, orange, red, and magenta bounding boxes represent the immature, semi-mature, mature, and defective categories, respectively.

**Figure 9:**
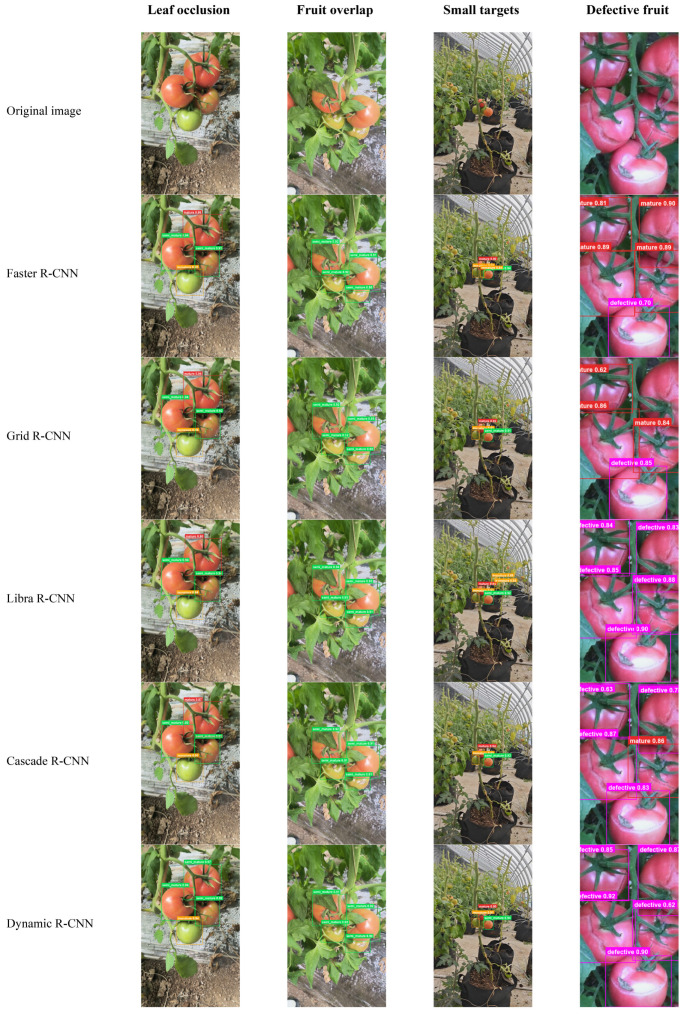
Detection results of two-stage detectors. Green, orange, red, and magenta bounding boxes represent the immature, semi-mature, mature, and defective categories, respectively.

To provide a qualitative comparison, representative test images were selected for visualization analysis, as shown in Figure 8 and Figure 9. Figure 8 includes four cases: leaf occlusion, fruit overlap, small targets, and defective fruits. The compared one-stage and end-to-end detectors identified most visible fruits, but missed detections, incomplete localization, or category confusion still occurred in some occluded, overlapping, and small-target regions. In comparison, TMAF-YOLO showed more complete target coverage and more stable category recognition in the selected examples. As shown in Figure 9, the two-stage detectors also localized most visible targets, although their predictions were still affected by occlusion, overlap, complex backgrounds, and small targets. Together with Table 3, these results indicate that TMAF-YOLO achieved a favorable balance between detection performance and inference efficiency.

**Figure 10:**
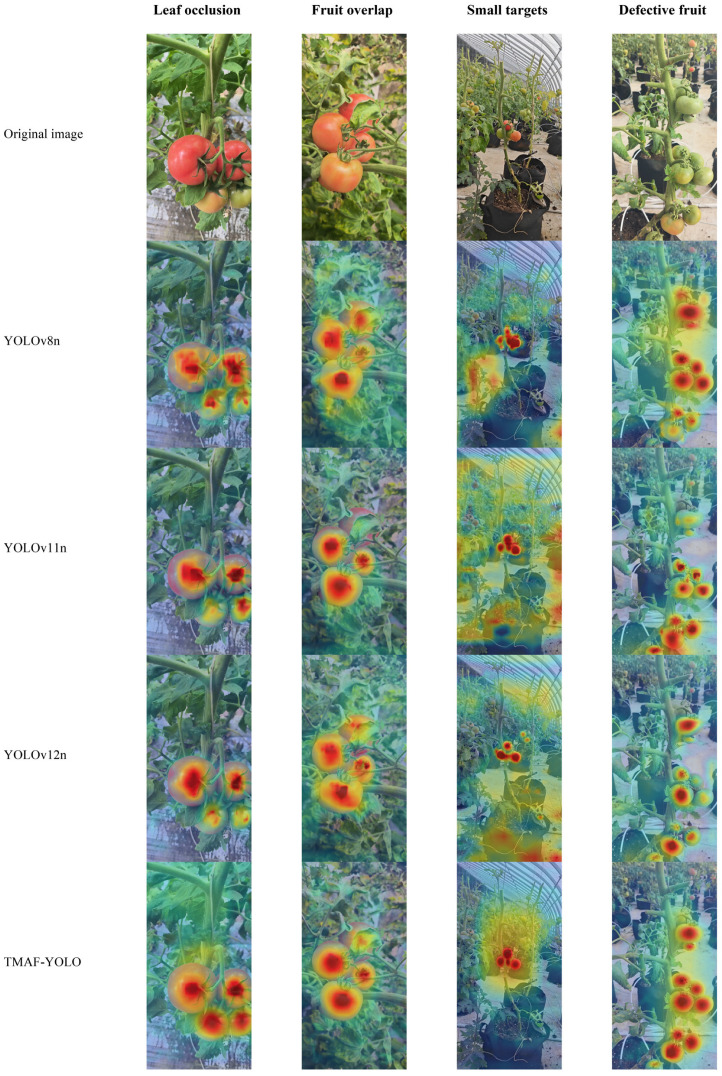
Heatmap visualization of YOLO-series models.

To further examine the spatial responses of the YOLO-based models, heatmap visualization was performed, as shown in Figure 10. High-response regions indicate image areas receiving greater attention during detection. The compared models generally responded to tomato regions, although some activation remained distributed over leaves, stems, fruit edges, or background areas. TMAF-YOLO showed relatively concentrated responses on visible fruit regions in the leaf-occlusion, fruit-overlap, and defective-fruit examples, while the response in the small-target scene remained relatively broad because of the limited target scale and complex background. Overall, these results suggest that the proposed feature-fusion structure helps suppress irrelevant background responses and strengthen fruit-related feature representation.

### 3.3. Ablation Study

To verify the effectiveness of each improved module, ablation experiments were conducted using YOLOv8n as the baseline model. The experiments were performed under the same dataset, training strategy, and experimental environment. The experimental settings included the original YOLOv8n, YOLOv8n + LGhostConv, YOLOv8n + TMAF, YOLOv8n + LGhostConv + TMAF, and the final TMAF-YOLO. The evaluation results on the test set are shown in Table 4.

As shown in Table 4, after introducing LGhostConv, the number of parameters decreased from 3.012 M to 2.729 M. FLOPs decreased from 8.2 G to 7.7 G, and FPS increased to 226.283. These results indicate that LGhostConv can reduce redundant computation and improve inference speed. After introducing TMAF alone, mAP50 increased from 0.916 to 0.941, and mAP50-95 increased from 0.744 to 0.762. This indicates that maturity-related feature representation was enhanced.

After adding MA-CB Focal Loss, the final TMAF-YOLO achieved Precision, Recall, mAP50, and mAP50-95 values of 0.889, 0.873, 0.954, and 0.776, respectively. Since this loss only acts on the classification branch during training, the number of parameters and computational cost remained the same as those of YOLOv8n + LGhostConv + TMAF. Compared with the original YOLOv8n, the inference time per image decreased from 6.493 to 4.784 ms, corresponding to a 26.3% reduction in visual detection latency. This reduction can shorten the waiting time for fruit recognition in an automated harvesting workflow, although it does not represent an equivalent reduction in the complete robotic harvesting cycle.

**Figure 11:**
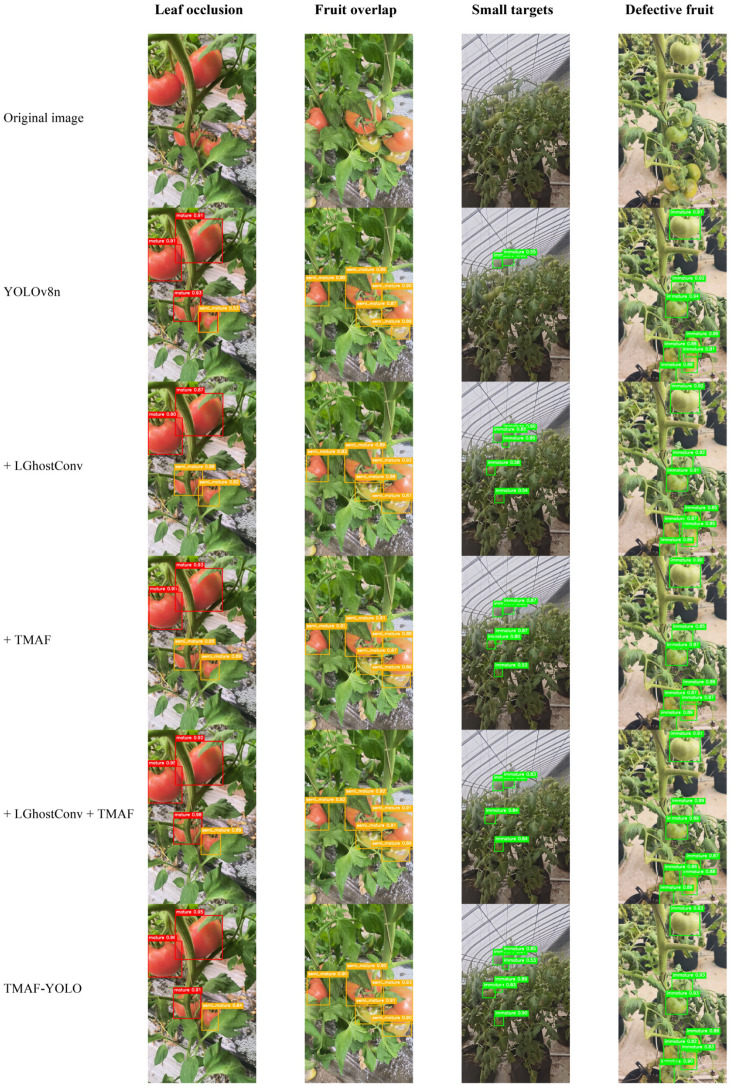
Detection results of ablation models. Green, orange, red, and magenta bounding boxes represent the immature, semi-mature, mature, and defective categories, respectively.

**Figure 12:**
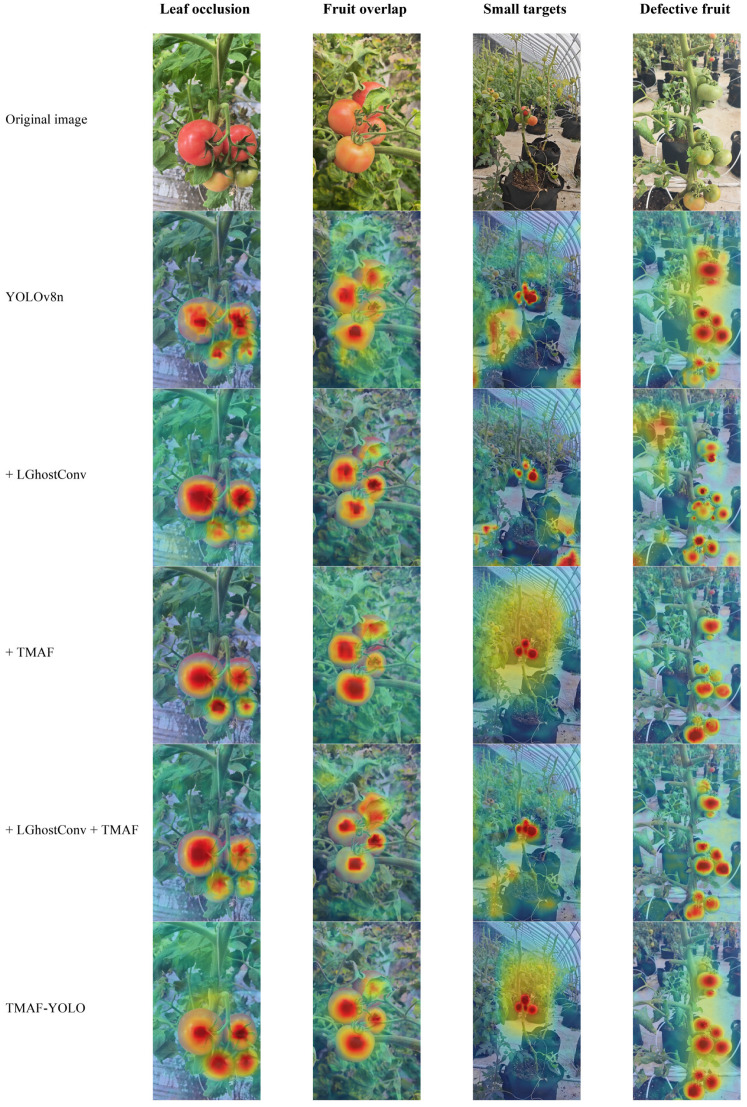
Heatmap visualization of ablation models.

To further examine the effects of the proposed modules, detection-result and heatmap visualizations were performed, as shown in Figure 11 and Figure 12. As shown in Figure 11, YOLOv8n still exhibited missed detections or unstable category predictions in some occluded, overlapping, and small-target regions. LGhostConv largely maintained the baseline detection performance. After TMAF was introduced, target coverage became more complete in some challenging regions. The final TMAF-YOLO generally produced more complete and stable detection results in the selected examples, particularly for small targets.

As shown in Figure 12, the responses of YOLOv8n were partly distributed over leaves, branches, or background regions in some scenes, whereas the models containing TMAF showed more concentrated responses on visible fruit areas. Together with the quantitative results in Table 4, these visualizations suggest that LGhostConv mainly contributes to complexity reduction and TMAF strengthens fruit-related feature representation, while the additional gain from MA-CB Focal Loss is reflected primarily in the quantitative results.

### 3.4. Discussion

#### 3.4.1. Methodological Significance and Practical Implications

The experimental results demonstrate that TMAF-YOLO achieves a favorable trade-off between detection accuracy and inference efficiency for greenhouse tomato maturity detection. The methodological contribution lies in the task-oriented integration of lightweight feature extraction, maturity-aware feature fusion, and classification loss optimization to jointly address fine-grained maturity discrimination and resource-efficient deployment. LGhostConv is introduced at selected downsampling positions to reduce redundant computation and model complexity while preserving useful feature information.

In addition, the TMAF module is placed before the detection heads to strengthen the representation of maturity-related features. Through local texture aggregation and channel-spatial recalibration, the model can better capture tomato color transitions, local surface textures, and occlusion boundaries. This is beneficial for distinguishing adjacent maturity stages, especially semi-mature and mature tomatoes with similar visual appearances. MA-CB Focal Loss further adjusts the contribution of different categories and hard samples during training. It reduces the dominance of easily classified samples and increases the learning attention given to confused maturity stages and the defective class.

Based on the harvesting rule defined in Section 2.1, the missed-detection rate for mature tomatoes, defined as the proportion of true mature fruits predicted as background, decreased from 5% to 2%. The potential mistaken-harvest rate for immature tomatoes, defined as the proportion of true immature fruits predicted as semi-mature or mature, decreased from 6% to 2%. Together with the 26.3% reduction in per-image inference time, these results indicate potential benefits in reducing missed detections of mature fruits, premature harvesting of immature fruits, and visual-processing delay.

#### 3.4.2. Limitations and Potential Improvements

This study still has several limitations. First, the dataset was mainly collected from greenhouse environments. Although it included common challenging conditions, such as leaf occlusion, fruit overlap, complex backgrounds, and different maturity stages, the data scale and scene diversity were still limited. Under sharp illumination changes, severe occlusion, or very small target conditions, missed detections and unstable classification may still occur. In future work, images from different greenhouses, shooting distances, illumination conditions, cultivars, and growth periods should be further collected to improve model generalization.

Second, tomato maturity changes continuously during fruit development. The visual boundary between semi-mature and mature tomatoes is not always clear. When only single RGB images are used, the model may be affected by illumination variation, fruit surface reflection, and partial occlusion. Future studies could introduce color space analysis, multiscale texture features, or time-series image information to better describe continuous maturity changes.

Finally, the proposed method is based on single-view RGB images, which cannot directly provide fruit depth, spatial position, or occlusion hierarchy. This limits the model when fruits are highly overlapped or severely covered by leaves. Multiview images, depth cameras, or lightweight three-dimensional perception methods could be considered in future work to improve spatial understanding in complex greenhouse scenes. However, no complete robotic harvesting experiment was conducted. The reported 26.3% reduction in per-image inference time therefore applies only to visual detection and should not be interpreted as an equivalent reduction in total harvesting time. Future work should evaluate harvesting time per fruit, harvesting success rate, and operational stability in real greenhouse robotic trials.

## 4. Conclusions

This study developed TMAF-YOLO, a lightweight tomato maturity detection model based on YOLOv8n. LGhostConv was introduced to reduce parameters and computational cost, while the TMAF module was designed to enhance maturity-related feature representation before the detection heads. In addition, MA-CB Focal Loss was used to improve model learning under class imbalance and hard-sample conditions.

The experimental results showed that TMAF-YOLO achieved mAP50 and mAP50-95 values of 0.954 and 0.776, respectively, while maintaining a lightweight structure and real-time inference capability. The ablation experiments further confirmed the contribution of LGhostConv, TMAF, and MA-CB Focal Loss. Compared with YOLOv8n, the visual detection latency was reduced by 26.3%. The confusion-matrix-based analysis further indicated lower missed-detection and potential mistaken-harvest rates for mature and immature tomatoes, respectively. These results demonstrate the potential of TMAF-YOLO to support efficient tomato maturity recognition and harvesting decision-making in greenhouse environments.

Future work will expand the dataset and evaluate embedded deployment and robotic harvesting performance under more diverse greenhouse conditions.

## Figures and Tables

**Table 1:** Distribution of images and annotated tomato instances before data augmentation.

Dataset Subset	Images	Immature	Semi-Mature	Mature	Defective	Total Instances
training	503	916	628	243	206	1993
validation	63	136	83	29	19	267
test	63	118	77	32	18	245
total	629	1170	788	304	243	2505

**Table 2:** Normalized confusion matrices of YOLOv8n and TMAF-YOLO.

	Predicted/True	Immature	Semi-Mature	Mature	Defective	Background
(a) YOLOv8n	immature	0.87	0.03	0.00	0.06	0.43
	semi-mature	0.06	0.86	0.14	0.00	0.23
	mature	0.00	0.05	0.79	0.02	0.12
	defective	0.00	0.01	0.02	0.89	0.20
	background	0.07	0.06	0.05	0.03	0.00
(b) TMAF-YOLO	immature	0.91	0.03	0.00	0.03	0.47
	semi-mature	0.02	0.91	0.10	0.00	0.20
	mature	0.00	0.04	0.86	0.04	0.08
	defective	0.00	0.00	0.02	0.90	0.25
	background	0.07	0.02	0.02	0.03	0.00

**Table 3:** Performance comparison of different detection models.

Model	Precision	Recall	mAP50	mAP50-95	Params (M)	FLOPs (G)	FPS	Inference Time (ms)
YOLOv8n	0.858	0.836	0.916	0.744	3.012	8.2	154.012	6.493
YOLOv11n	0.818	0.848	0.909	0.750	2.591	6.5	199.481	5.013
YOLOv12n	0.838	0.824	0.897	0.728	2.569	6.5	140.763	7.107
DETR	0.787	0.875	0.872	0.607	41.556	77.1	23.937	41.777
DINO	0.859	0.834	0.899	0.704	47.393	218.6	30.164	33.152
Faster R-CNN	0.846	0.871	0.882	0.661	41.364	61.7	24.696	40.493
Grid R-CNN	0.859	0.873	0.870	0.673	64.700	154.0	19.388	51.577
Libra R-CNN	0.867	0.828	0.872	0.654	41.270	62.4	24.960	40.065
Cascade R-CNN	0.856	0.861	0.885	0.693	69.161	89.5	18.875	52.980
Dynamic R-CNN	0.822	0.885	0.862	0.641	41.364	61.7	24.539	40.751
TMAF-YOLO	0.889	0.873	0.954	0.776	2.647	7.3	209.030	4.784

**Table 4:** Ablation results of different improved modules.

Model	Precision	Recall	mAP50	mAP50-95	Params (M)	FLOPs (G)	FPS	Inference Time (ms)
YOLOv8n	0.858	0.836	0.916	0.744	3.012	8.2	154.012	6.493
YOLOv8n + LGhostConv	0.868	0.820	0.920	0.749	2.729	7.7	226.283	4.419
YOLOv8n + TMAF	0.875	0.855	0.941	0.762	2.930	7.8	194.175	5.150
YOLOv8n + LGhostConv + TMAF	0.880	0.861	0.949	0.768	2.647	7.3	207.340	4.823
TMAF-YOLO	0.889	0.873	0.954	0.776	2.647	7.3	209.030	4.784

## Data Availability

Data will be made available on request.
